# Effects of Moderate Aerobic Exercise Training on Hemorheological and Laboratory Parameters in Ischemic Heart Disease Patients

**DOI:** 10.1371/journal.pone.0110751

**Published:** 2014-10-27

**Authors:** Barbara Sandor, Alexandra Nagy, Andras Toth, Miklos Rabai, Bela Mezey, Arpad Csatho, Istvan Czuriga, Kalman Toth, Eszter Szabados

**Affiliations:** 1 1^st^ Department of Medicine, University of Pecs, School of Medicine, Pecs, Hungary; 2 Department of Behavioural Sciences, University of Pecs, School of Medicine, Pecs, Hungary; Maastricht University Faculty of Health, Medicine, and Life Sciences, Netherlands

## Abstract

**Background and Design:**

In this study we set out to determine the effects of long-term physical training on hemorheological, laboratory parameters, exercise tolerability, psychological factors in cardiac patients participating in an ambulatory rehabilitation program.

**Methods:**

Before physical training, patients were examined by echocardiography, tested on treadmill by the Bruce protocol, and blood was drawn for laboratory tests. The enrolled 79 ischemic heart disease patients joined a 24-week cardiac rehabilitation training program. Blood was drawn to measure hematocrit (Hct), plasma and whole blood viscosity (PV, WBV), red blood cell (RBC) aggregation and deformability. Hemorheological, clinical chemistry and psychological measurements were repeated 12 and 24 weeks later, and a treadmill test was performed at the end of the program.

**Results:**

After 12 weeks Hct, PV, WBV and RBC aggregation were significantly decreased, RBC deformability exhibited a significant increase (p<0.05). Laboratory parameters (triglyceride, uric acid, hsCRP and fibrinogen) were significantly decreased (p<0.05). After 24 weeks the significant results were still observed. By the end of the study, IL-6 and TNF-α levels displayed decreasing trends (p<0.06). There was a significant improvement in MET (p<0.001), and the BMI decrease was also significant (p<0.05). The vital exhaustion parameters measured on the fatigue impact scale indicated a significant improvement in two areas of the daily activities (p<0.05).

**Conclusions:**

Regular physical training improved the exercise tolerability of patients with ischemic heart disease. Previous publications have demonstrated that decreases in Hct and PV may reduce cardiovascular risk, while a decrease in RBC aggregation and an increase in deformability improve the capillary flow. Positive changes in laboratory parameters and body weight may indicate better oxidative and inflammatory circumstances and an improved metabolic state. The psychological findings point to an improvement in the quality of life.

## Introduction

In the past few decades, mortality due to coronary artery disease (CAD) has decreased substantially in the industrialized countries thanks to the improved medical care, but it remains the leading cause of death worldwide. In addition to the optimal pharmacological therapies and modern revascularization procedures, a number of preventive strategies have been created with a view to the further reduction of the morbidity and mortality of CAD [1].

The recent guidelines of the European Society of Cardiology (ESC) and the American Heart Association/American College of Cardiology (AHA/ACC) indicate that physical activity has a pivotal role in the primary prevention in healthy subjects [1], and moreover it reduces the all-cause and cardiovascular (CV) mortality too [1], [2], [3], [4], [5], [6], [7]. Moderate aerobic exercise training in patients with CAD improves myocardial perfusion, muscular endurance and psychosocial well-being leading to enhanced flexibility, ameliorated symptoms, better cardiorespiratory fitness and a reduced CV risk [1]. Training from 2.5 to 5 hours a week can result in a 20–30% CV and all-cause mortality risk reduction. In summary, moderate physical activity reduces the CV risk in a dose-dependent manner in both male and female healthy subjects, and even in patients with known CAD [1]. Physical activity conducted by physiotherapists and supervised by a cardiologist in a cardiac rehabilitation (CR) program is an excellent possibility for secondary prevention, where effective risk factor management can be achieved through long-term life style changes [1].

Impaired hemorheological parameters, including reduced erythrocyte deformability and increased erythrocyte aggregation, may have a deleterious effect on the vascular system leading to the development of various CV, cerebrovascular and peripheral arterial diseases [8], [9], [10], [11], [12]. Whereas publications from the last 25 years have clearly revealed a relationship between hemorheological factors and physical training, those studies involved healthy volunteers or a small numbers of CV patients participating in short-term (10–12 weeks) exercise training. Furthermore, the possible connections between hemorheology and long-term, moderate aerobic physical activity have not been investigated in a relatively large population with ischemic heart disease.

Our study had the aim of determining the beneficial effects of aerobic physical training on ischemic heart disease patients participating in a long-term (24 weeks) ambulatory CR program.

## Methods

79 non-smoker patients with stable ischemic heart disease (39 males and 40 females, mean age: 65.3±5.68 years) were selected for the study; their co-morbidities are presented in Table 1. The patients received their medication in accordance with current guidelines for the secondary prevention of CAD [13] and with their co-morbidities (Table 2). Modifications in either agent or dose were not made during the trial. Patients with an ejection fraction <40%, and MET <5 or a significant ST depression during a treadmill-based exercise tolerance test using the Bruce protocol were excluded from the study [14], [15]. ST depression was accepted significant in thoracic leads by 1 mm and in limb leads by 2 mm. There were neither arrhythmias nor AV blocks detected among our patients.

**Table 1:** Characteristics of the study population.

population characteristic		number of patients	%
ischemic heart disease	myocardial infarction	32/79	40.51
	previous CABG	35/79	44.3
	previous PCI	18/79	22.78
	proven by coronary angiography or coronary CT	18/79	22.78
hypertension		71/79	89.87
diabetes mellitus		24/79	30.38

**Table 2:** Medication during the 24 week long physical training.

medication	number of patients	%
cholesterol lowering drugs	70/79	88.61
antiplatelet drugs	79/79	100
β-blocker	59/79	74.68
RAAS inhibitor	65/79	82.28

The investigation was approved by the Regional Ethics Committee (licence number: 4378) of the University of Pecs and written informed consent was signed by all subjects.

### Study design

At baseline, the following measurements were performed: psychological tests, resting electrocardiography (ECG), resting echocardiography (Table 3), treadmill-based exercise tolerance testing using the Bruce protocol, clinical chemistry (fasting total cholesterol, triglyceride, high-density lipoprotein (HDL), low-density lipoprotein (LDL), uric acid, hsCRP, fasting glucose, total plasma protein, albumin, blood cell counts, fibrinogen, cytokines (TNF-α and IL-6) and hemorheological measurements (Hct, whole blood viscosity (WBV), the ratio Hct/WBV, plasma viscosity (PV), red blood cell aggregation and red blood cell deformability).

**Table 3:** Results of the echocardiographycal measurements at baseline.

echocardiographical parameters	
ejection fraction (%)	59.92±8.41
left ventricle end-systolic diameter (mm)	50.09±6.7
left ventricle end-diastolic diameter (mm)	31.71±5,51

N = 79, values are means ± SD.

The patients participated in a 24-weeks physical training program lasting for 1 hour 3 times weekly, designed and conducted by a physiotherapist and supervised by a cardiologist. After 12 weeks, the hemorheological measurements, clinical chemistry (except cytokines) and psychological tests were repeated. At the end of the 24 weeks, the resting ECG measurements, the treadmill tests with the Bruce protocol, the clinical chemistry, the hemorheological measurements and the psychological tests were repeated.

### Aerobic exercise training program

The present aerobic exercise training program was preceded and ended with blood pressure and pulse measurements. The patients began with warm-up exercises (breathing exercises, and stretching of the large joints) for 5–10 minutes. In the second phase, they participated in a moderate-intensity training. Intensity was defined as 50–70% of peak VO_2_ (starting at 50% and gradually increasing to 70% of VO_2max_). The intensity was assessed by the Borg scale (13–15/20) [16] and pulse measurements. The training involved static (exercises with medicine ball, half-squats, toe raisis, body flexions) and dynamic (walking, jogging, ball games e.g. basketball, football) exercise elements. The aerobic phase lasted 35–40 minutes. Finally, relaxation exercises were performed (stretching and breathing exercises) for 10 minutes.

### Blood collecting

Blood samples were obtained from the antecubital vein at baseline, after 12 weeks and after 24 weeks. The blood was collected into two lithium heparin-coated (12 ml), one clot activator-coated and gel-containing (5 ml), one potassium EDTA-coated (3 ml) and one sodium fluoride and potassium oxalate-coated (2 ml) Vacutainer tube with a 21-gauge Eclipse Blood Collection butterfly needle set, using a minimal tourniquet.

### Hemorheological measurements

Hemorheological measurements were performed within 2 hours after blood sampling. Hct was measured by using a micro-Hct centrifuge (Haemofuge Heraeus Instr., Germany). WBV and PV were determined at a shear rate of 90 s^−1^ with a Hevimet 40 capillary viscometer (Hemorex Ltd., Budapest, Hungary). Plasma was prepared by a 10-minute centrifugation of whole blood at 1500 g. Measurements were made at 37°C. The ratio Hct/WBV was utilized to characterize RBC oxygen transport effectiveness [11].

Red blood cell aggregation was measured with LORCA (Laser-assisted Optical Rotational Cell Analyzer; R&R Mechatronics, Hoorn, The Netherlands) [17], [18] aggregometer, using blood samples with standard 40% Hct. RBC aggregation index (AI) was determined at 37°C via syllectometry (i.e., laser backscatter versus time). The RBC disaggregation threshold (γ), i.e., the minimal shear rate needed to prevent RBC aggregation or to breakdown existing RBC aggregates, was determined using a re-iteration procedure. Measurements were made at 37°C.

Erythrocyte deformability was characterized with a LORCA ektacytometer [19] at 37°C, which provided nine values of elongation index (EI) in the shear stress range from 0.3 to 30 Pa. The deformability results were analyzed by means of the Lineweaver-Burke nonlinear equation, with calculation of the maximal EI (EI_max_) at infinite shear, and the shear stress value (SS_1/2_) required for half of this maximal elongation [20]. For deformability measurements, blood samples were suspended in a highly viscous (32.6 mPas) polyvinylpyrrolidone solution.

### Cytokine measurements

Cytokines were determined with an automated chemiluminescence immunoassay system (Immulite 1000, Siemens). For TNF-α, a solid-phase chemiluminescent immunometric assay (cat. no. LKNF1), and for IL-6, a solid-phase chemiluminescent sequential immunometric assay (cat. no. LK6P1) was used. Master calibration and bi-level cytokine controls were applied during the runs.

### Psychological surveys

In order to examine the effects of the 24-week physical training on the patients' subjective experience with fatigue, we applied the Fatigue Impact Scale (FIS) [21], [22]. The FIS consists of 40 items which evaluate the impact of fatigue on three aspects of daily life: physical (10 items), cognitive (10 items) and psychosocial (20 items) functions. In addition, we monitored patients' depression severity by the short version of the Beck Depression Inventory (9 items).

### Statistics

Data are shown as means ± SD. Differences were evaluated by a one-way repeated ANOVA statistical test (Tamhane post-hoc test) after using the Kolmogorov–Smirnov test to check on the normality of the data distribution. Multivariate linear regression and stepwise analyses of the data were performed with regard to differences between the baseline and the 24-week MET values for whole blood and plasma viscosity, LORCA aggregation index, LORCA erythrocyte deformability at 5,33 Pa shear stress and BMI.

A sample size and power analysis was performed for the overall population using PS program version 3.1.2. For the sample size of n = 79 patients needed to detect a true difference of δ = 1.94 in MET with 92.6% power, where type I error probability is α = 0.05.

The psychological data revealed a significant deviation from the normal distribution, and; the nonparametric Friedman test was therefore applied to analyze potential changes in psychological functioning. The analyses of the psychological data was restricted to those patients who had no missing surveys and gave no indication of moderate to severe depression at any of the three measurements. Five patients indicated moderate to severe depression during the rehabilitation period, and six of them had missing surveys. These patients were excluded from the psychological data analyses, which included 68 patients' data (86% of the total sample).

Significance level was defined as p<0.05. SPSS statistical software, version 11.0.1. was used to conduct descriptive analyses and to describe the sample.

## Results

As concerns the hemorheological results, the Hct displayed a decreasing tendency during the investigated period, while the WBV exhibited a significant reduction (p<0.05), resulting in a significantly increased Hct/WBV ratio (p<0.05). The PV was significantly decreased after 12 weeks and remained significantly lower relative to the baseline at the end of the program (p<0.001) (Table 4). The red blood cell aggregation parameters of LORCA aggregometer likewise demonstrated significant reductions. The LORCA parameter (AI) decreased significantly during the 24-week training program (p<0.05) (Table 5), while the LORCA EIs of erythrocyte deformability increased significantly (p<0.001), supported by the Lineweaver-Burke nonlinear equation analyses showing a significantly higher EI_max_ and a significantly lower SS_1/2_ (Table 6).

**Table 4:** Changes in certain hemorheological parameters (hematocrit (Hct), whole blood viscosity (WBV), plasma viscosity (PV) and the Hct/WBV ratio) after the 12- and 24-week ambulatory exercise training of ischemic heart disease patients.

hemorheological parameters	mean ± SD	p value
hematocrit (%)	44.18±4.56 vs. 42.98±6.0 vs. 42.13±7.7	0.11
whole blood viscosity (mPas)	4.35±0.59 vs. 4.26±0.62 vs. 4.08±0.77	0.03
plasma viscosity (mPas)	1.27±0.22 vs. 1.18±0.2 vs. 1.14±0.23	0.001
Hct/WBV (1/mPas)	10.2±0.63 vs. 10.25±1.18 vs. 10.33±1.7	0.04

N = 79; values are baseline, 12 week and 24 week means ± SD. Levels of significance and p values are also shown.

**Table 5:** Changes in erythrocyte aggregation measured with LORCA aggregometers and with standard 40% Hct after the 12- and 24-week ambulatory exercise training of ischemic heart disease patients.

hemorheological parameters	mean ± SD	p value
LORCA AI	65.26±5.9 vs. 64.04±6.9 vs. 63.9±5.4	0.01
LORCA γ (1/s)	128.6±37.4 vs. 116.17±41.4 vs. 123.1±37.9	0.07

N = 79; values are baseline, 12 week and 24 week means ± SD. Levels of significance and p values are also shown.

**Table 6:** Changes in erythrocyte deformability parameters EI_max_ and SS_1/2_ calculated by the Lineweaver-Burke nonlinear equation after the 12- and 24-week ambulatory exercise training of ischemic heart disease patients.

hemorheological parameters	mean ± SD	p value
EI_max_	0.669±0.16 vs. 0.674±0.01 vs. 0.673±0.1	0.04
SS_1/2_ (mPas)	2.104±0.57 vs. 1.831±0.2 vs. 1.769±0.4	0.0004

N = 79; values are baseline, 12 week and 24 week means ± SD. Levels of significance and p values are also shown.

The clinical chemistry parameters relating to uric acid, triglycerides, hsCRP and fibrinogen decreased significantly during the training period (Table 7).

**Table 7:** Changes in clinical chemistry after the 12- and 24-week ambulatory exercise training of ischemic heart disease patients.

clinical chemistry	mean ± SD	p value
glucose (mmol/l)	5.16±2.9 vs. 5.19±1.7 vs. 4.80±2.0	0.48
uric acid (umol/l)	336.39±55.85 vs. 327.17±82.68 vs. 312.88±95.1	0.006
total cholesterol (mmol/l)	4.99±1.1 vs. 4.87±1.3 vs. 4.61±1.4	0.17
HDL (mmol/l)	1.40±0.3 vs. 1.39±0.4 vs. 1.33±0.5	0.53
LDL (mmol/l)	2.57±0.7 vs. 2.50±0.8 vs. 2.46±0.9	0.69
triglyceride (mmol/l)	1.70±0.8 vs. 1.56±0.7 vs. 1.46±0.8	0.017
hsCRP (mg/l)	6.02±2.3 vs. 2.98±2.4 vs. 3.04±2.5	0.0008
fibrinogen (g/l)	3.28±1.3 vs. 3.03±0.5 vs. 3.15±0.8	0.03
IL 6 (pg/ml)	3.95±1.87 vs. 1.88±1.24	0.06
TNF α (pg/ml)	11.53±5.9 vs. 10.21±2.95	0.11

N = 79; values are baseline, 12 week and 24 week means ± SD. Levels of significance and p values are also shown.

The cytokine measurements did not indicate a significant decrease (p<0.05), but only a falling trend as compared with the baseline values (Table 7).

As expected, the functional capacity described by the MET significantly improved (p<0.001), and the treadmill time also increased significantly, by 17.4% (p<0.001), during the training program. Moreover, the patients lost weight, with the body mass index (BMI) undergoing a significant decrease during the trial (p<0.001), however lean body mass index calculated by the Hume formula [23], showed a slight but not significant increase at the end of the 24 week (Table 8).

**Table 8:** Changes in exercise tolerability parameters (MET and treadmill time) and BMI as well as lean body mass index after the 24-week ambulatory exercise training of ischemic heart disease patients.

treadmill	mean ± SD	p value
MET	7.93±2.4 vs. 9.87±5.7	<0.001
treadmill time (min)	7.68±2.17 vs. 9.02±2.18	0.001
BMI (kg/m^2^)	28.16±5.53 vs. 25.45±4.1	<0.001
lean body mass index (kg)	51.71±6.6 vs. 52.78±9.4	0.09

N = 79; values are baseline and 24 week means ± SD. Levels of significance and p values are also shown.

For the Δ values between the baseline and 24-week measurements were calculated from every parameter showing a significant difference to the baseline. The Δ parameters, which were in positive or negative correlation to the MET were used for the multivariate linear regression analyses: five independent variables were investigated in association with the difference in MET (ΔMET was regarded as the dependent variable). Regression analysis showed that the predictive model provided a good fit to the data with a significant F value (F(5), p<0.001), and the five predictors explained 76% of the difference in MET values (R^2^ = 0.76). The results in Table 9 indicated that the change in red blood cell aggregation index (LORCA) (standardized β = −0.337), WBV (standardized β = 0.406) and red blood cell deformability at 5,33 Pa shear stress (standardized β = −0.197) values were significant independent variables of the regression model and the most strongly related to the variation of the MET values. Furthermore, the analyses revealed a strong independent predictive association between the ΔAI (LORCA), ΔWBV as well as Δdeformability and the dependent variables.

**Table 9:** Results of the multivariate linear regression analyses after the 24-week ambulatory exercise training of ischemic heart disease patients; N = 79.

predictor (pre and post data)	standardized β	p value (p<0.05)
Δ whole blood viscosity (4.35 vs. 4.08)	0.406	0.001
Δ plasma viscosity (1.27 vs. 1.14)	−0.025	0.84
Δ LORCA aggregation index (66.67 vs. 62.25)	−0.337	0.011
Δ BMI (28.16 vs. 25.45)	−0.197	0,08
Δ elongation index (5.33Pa) (0.490 vs. 0.510)	−0.157	0.015

Although gender specific subgroup analyses was made regarding hemorheological, laboratory chemistry and exercise tolerance parameters, but no differences were observed. Data are not shown.

In the course of the study, there was no drop out, and no noteworthy CV event or unplanned hospitalization occurred. Analysis of the FIS data revealed a significant decline in the symptoms of fatigue in the physical [chi^2^(2)  = 6.12, p<0.05], the psycho-social [chi^2^(2)  = 7.09, p<0.05] and in the cognitive domain [chi^2^(2)  = 8.85, p<0.05]. More specifically, patients' perception of their physical, cognitive, and social functional limitations caused by fatigue declined significantly over the course of the physical training period.

## Discussion

The fundamental problem of CAD patients can not be solved completely via revascularization techniques (percutaneous coronary intervention or a coronary artery bypass graft), effective and long-term lifestyle changes are at least as vital as other therapeutic procedures [24]. Recent studies such as EuroAction [25] and GOSPEL [26] have indicated that regular long-term physical activity results in more benefit than short-term training programs as regards the prognosis of cardiac patients. A physical training program is strongly recommended by the ESC and the AHA/ACC as well [1], [2], [3].

Aerobic exercise training is defined as a sub-category of physical activity in which planned, structured, and repetitive bodily movements are performed to maintain or improve physical fitness [1]. According to the recommendations of ESC guidelines regarding physical activity and CAD prevention [1], [2], patients with previous acute myocardial infarction, CABG, PCI, stable angina pectoris, or stable chronic heart failure should undergo exercise training (I (A) strong evidence) [27], which should be performed at least 30 minutes long (preferably 45–60 minutes) and 3–5 times weekly, in form of an aerobic exercise training (I (B) evidence) [1], [2], at 70–85% of the peak heart rate [2] or 40–60% of heart rate reserve [2] or 10/20–14/20 of the Borg Scale [16].

It is a long revealed fact that impaired hemorheological factors are CV risk factors, and the improvement of these could result in lower CV risk and mortality [8]–[12]. The triphasic association of hemorheology and physical exercise is as well identified by now. Still, the possible connections between hemorheology and long-term aerobic physical training in a relatively large ischemic heart disease population have not been investigated previously. A systematic literature search in PubMed with the keywords hemorheology, physical activity, physical exercise, cardiovascular diseases and atherosclerosis, identified only 14 nonrandomized controlled studies from the past 25 years in which original data were used to determine changes in hemorheological parameters, mostly in healthy volunteers and athletes (9 publications), but also partially in patients with CV diseases (5 publications), participating in short-term (10–12 weeks) exercise training [28] (Tables S1 in File S1, Table S2 in File S2, Table S3 in File S3). Investigations involving healthy volunteers have proven that short-term physical activity has acute effects on the hemorheological parameters, including increases in Hct and WBV due to the fluid shift, water loss and release of sequestered red blood cells from the spleen [29]–[31]. In contrast, long-term training causes autohemodilution, resulting in reduction of PV and WBV [29]. On the other hand, the findings of red blood cell aggregation and deformability studies are not concordant. Some investigations dealing with long-term physical activity performed by healthy volunteers, suggested the deterioration of rheological factors [32]–[34], whereas others reached the opposite conclusion [35]–[37]. These discrepancies might be explained by differences in training periods (short,- or long-term), study designs [29], [31], [35], methods [32], [33], [36], [37], the selected populations [31], [33], [34] and the exercise performed (cycling vs. running) [29], [31].

Only few studies have focused on the effects of exercise on hemorheological factors in CV patients [28]. Several investigations have shown that acute training evokes increases in PV and fibrinogen [38]. *Levine et al.* were unable to demonstrate any hemorheological effects after a 10-week CR training [39], but *Church et al.* reported reductions in WBV and PV after a 12-week CR program [40]. In our present study, we investigated whether we could demonstrate any hemorheological changes in ischemic heart disease patients participating in a 24-week ambulatory CR training program. The results pointed to a slight decrease in Hct, while significant decreases were observed in WBV and PV, resulting in a significantly increased Hct/WBV ratio. The red blood cell aggregation indices and the measured deformability parameters were also significantly enhanced at the end of the training program.

Although the blood flow of the coronary vessel system is primarily determined by hemodynamic factors (i.e. continuous changes in flow, extravascular pressure, perfusion pressure and shear rate during a cardiac cycle), under certain conditions (e.g. a flow decrease caused by vessel stenosis, especially in ischemic heart disease patients) the role of rheological parameters becomes important. The observed beneficial changes in the macrorheological parameters presented in this investigation (e.g. Hct and viscosity) presumably reduce the CV risk of ischemic heart disease patients [11], [12], [41], [42].

In addition to the positive findings among macrorheological parameters, microrheological changes could also have a pivotal role in the development of better physical fitness. The diameter of the narrowest capillaries in the body (3–5 µm), found in the myocardium, is appreciably less than the resting diameter of a normal red blood cell (7–8 µm), which highlights the importance of the microrheological parameters (e.g. erythrocyte aggregation and deformability). Decreased red blood cell aggregation and improved deformability observed in our trial support capillary flow, especially in patients suffering from ischemic heart disease. In the background of increased deformability we could assume recently published data, which have stated that aerobic training with low lactate levels (as aerobic exercise) could enhance RBC deformability [43], which together with an increased Hct/WBV ratio indicate a better RBC oxygen transport effectiveness leads to a better oxygen supply of the myocardiocytes, and the working muscles (43, 44). According to a lately observed phenomenon, cerebral and muscle tissue oxygenation indicis (TOI) measured by NIRS are positively correlated with Hct/WBV ratio in sickle-cell patients (SC and SS patients) comparing to healthy volunteers, which could also support our findings [45]. Thus, our results may suggest that cardiac patients could achieve a condition of “hemorheological fitness” characterized by improved tissue perfusion, better oxygen delivery and lower vascular resistance [28], [43], [44], [45] by participating in a physical training program for 24 weeks.

Furthermore, all hemorheological alterations may also contribute to the better exercise tolerability proved by the treadmill test parameter MET and the treadmill time. Moreover, the multivariate linear regression analyses showed that changes in red blood cell deformability, aggregation and WBV are independent predictors of the positive changes in MET. These results are in accordance with the above mentioned findings about improving RBC deformability and Hct/WBV ratio since they have a pivotal role in the enhancement of capillary blood flow, as well as in the oxygen supply of the myocardiocytes and the working muscles during exercise [43], [44], [45]. Beneath better physical tolerance, the improvement of MET by 1 value could reduce the risk of all-cause and CV mortality by 13% and 15%, respectively [46], [47].

Of the clinical chemistry indicators, uric acid, triglyceride, hsCRP and fibrinogen underwent significant decreases during our study. Although we are aware that these biomarkers are considered to display only low specificity in a CV risk assessment and are easily influenced by common inflammatory diseases, our data suggest that regular long-term physical activity might exert a favorable effect on the inflammation status of patients with CAD. Further overproduction of proinflammatory cytokines such as IL-6 or TNF-α could be a marker of chronic inflammation leading to provoked and accelerated atherosclerosis [48], [49], with a higher risk of CV events and mortality [50]. Our data demonstrated an almost significant decreases in IL-6 and TNF-α levels, suggesting that a longer training program might be required to achieve significant reductions in these parameters.

Interestingly, no significant change was observed in the fasting glucose. Our findings in case of glucose levels are supported by recent publication; authors did not observed any effect on the glycemic control caused by exercise training in 16 aged, female patients with type 2 diabetes mellitus neither with weekly 2 times for 60 minutes nor with weekly 4 times for 30 minutes [51]. Although the same paper described the beneficial hemorheological effects (decreased RBC aggregation and increased RBC deformability) of the mentioned training protocol [51].

However total cholesterol and LDL cholesterol levels did not change during the trial, triglyceride and uric acid levels decreased significantly by the end of the training program. It is well known that hypertriglyceridemia is a significant independent CV risk factor [52], [53] and a recently published metaanalysis concluded that an elevated serum uric acid level should be considered as a risk factor for CV mortality [54]. The significant reductions in triglyceride and uric acid levels in our study may reflect a better metabolic state evoked by regular physical activity.

Beside laboratory parameters, both overweight and obesity are associated with an elevated risk of death in CAD and of all-cause mortality [55]–[57]. The BMI was significantly decreased by the end of the 24-week exercise training in our study. Although the results of the multivariate linear regression model indicated that the positive change in functional capacity is not influenced by the reduction in BMI. Accordingly, the better physical exercise tolerance can not be explained merely by the decreases in BMI and obesity. The beneficial effects of the physical activity generally on all the measured CV risk factors, including the hemorheological factors, must also be involved.

Moreover, CAD is often accompanied by an increased subjective feeling of fatigue (i.e. a feeling of physical tiredness and a lack of energy) and this might have a serious detrimental impact on a wide variety of everyday functions, including physical, mental and social functioning [58]. Our psychological results showed a significant amelioration as concerns the FIS physical and psycho-social aspects, indicating an improvement in the quality of life among our ischemic heart disease patients.

Our study was designed as a self-control study, as our working group have already proved the worsening of the hemorheological factors in patients with stable CAD, even if these patients have received correct drug therapy, but have not attended at any CR program [41], [42]. Kesmarky et al. have observed significant deterioration in WBV and Hct as well as in fibrinogen levels, along with an increase of PV, 6 months after a percutaneous coronary angioplasty [41]. In accordance, Marton et al. described the same significant increase in WBV, Hct and PV in ischemic heart disease patients 1 year after their myocardial infarction [42].

## Conclusions

Our study has revealed new data regarding the effects of a long-term ambulatory CR training program on stable CAD patients. Besides the anticipated improvement in functional capacity and the reduction in BMI, the regular, moderate-intensity, long-term physical activity led to favorable hemorheological changes, decreased level of inflammation and improvements in certain metabolic parameters, such as the triglyceride and uric acid contents, suggesting that these parameters may play important part in the positive effects of regular physical activity in patients with CAD.

## Supporting Information

Table S1 **Publications relating to hemorheological alterations induced by acute exercise training programs in healthy volunteers.**
(DOC)

Table S2 **Publications relating to hemorheological alterations induced by short-term exercise training programs in healthy volunteers.**
(DOC)

Table S3 **Publications relating to hemorheological alterations induced by long-term exercise training programs in patients with cardiovascular diseases.**
(DOC)
