# Supplementary material for: Effects of Moderate Aerobic Exercise Training on Hemorheological and Laboratory Parameters in Ischemic Heart Disease Patients
Source: PLoS One. 2014 Oct 27;9(10):e110751. doi: 10.1371/journal.pone.0110751 (PMC4210208; doi:10.1371/journal.pone.0110751)
Supplement: Table S1 — Publications relating to hemorheological alterations induced by acute exercise training programs in healthy volunteers. (DOC) [file pone.0110751.s001.doc]

Table S1.

| authors | year of publication | study duration | population | exercise | results |
| --- | --- | --- | --- | --- | --- |
| Neuhaus et al. | 1992 | before and after exercise | 8 healthy  athletes | marathon running | no hemorheological changes |
| Yalcin et al. | 2003 | before and after exercise | 10 healthy untrained men | heavy anaerobic exercise | red blood cell deformability and aggregation decreased but normalized 24 hours after exercise |
| Connes et al. | 2004 | before and after exercise | 20 healthy athletes | progressive exercise test | red blood cell deformability increased |
| Connes et. al | 2007 | before and after exercise | 13 healthy sportsmen | progressive and maximal exercise | red blood cell aggregation did not change |
| Tripette J. et al. | 2011 | before and after exercise | 9 healthy athletes | 10 km running with fluid intake *ad libitum* | no hemorheological changes |

Publications relating to hemorheological alterations induced by acute exercise training programs in healthy volunteers.
