# Supplementary material for: Effects of Moderate Aerobic Exercise Training on Hemorheological and Laboratory Parameters in Ischemic Heart Disease Patients
Source: PLoS One. 2014 Oct 27;9(10):e110751. doi: 10.1371/journal.pone.0110751 (PMC4210208; doi:10.1371/journal.pone.0110751)
Supplement: Table S2 — Publications relating to hemorheological alterations induced by short-term exercise training programs in healthy volunteers. (DOC) [file pone.0110751.s002.doc]

Table S2.

| authors | year of publication | study duration | population | exercise | results |
| --- | --- | --- | --- | --- | --- |
| Ernst et al. | 1987 | 3 months | untrained men | regular  training | RBC deformability increased, WBV and PV decreased |
| Brun et al. | 1998 | short- and long-term | healthy men | cycling | increased viscosity after short-term training but autohemodilution after long term exercise |
| Cakir-Atabek et al. | 2009 | 3 times weekly 6 week long | 14 healthy untrained men | resistance exercise training | RBC deformability and RBC decreased after 6 weeks but after training increased immediately |
| Kilic-Toprak et al. | 2011 | 3 times weekly 12 week long | 12 healthy untrained men | progressive resistance exercise training | RBC deformability increased, RBC aggregation did not change, WBV and PV decreased |

Publications relating to hemorheological alterations induced by short-term exercise training programs in healthy volunteers.
