# Supplementary material for: Effects of Moderate Aerobic Exercise Training on Hemorheological and Laboratory Parameters in Ischemic Heart Disease Patients
Source: PLoS One. 2014 Oct 27;9(10):e110751. doi: 10.1371/journal.pone.0110751 (PMC4210208; doi:10.1371/journal.pone.0110751)
Supplement: Table S3 — Publications relating to hemorheological alterations induced by long-term exercise training programs in patients with cardiovascular diseases. (DOC) [file pone.0110751.s003.doc]

**Table S3.**

| authors | year of publication | study duration | population | exercise | results |
| --- | --- | --- | --- | --- | --- |
| Ernst et al. | 1987 | 5 times weekly 8 week long | 22 patients  with claudication | standardized treadmill exercise | WBV, PV and RBC aggregation decreased, RBC filterability increased |
| Levine et al. | 1995 | 3 days weekly 10 week long | 15 patients with known CAD underwent CR | 30-40 minutes moderate-intensity exercise | no hemorheological changes |
| Reinhart et al. | 1998 | 1 hour daily 8 week long | 25 patients with post myocardial infarction and EF<40% | cycling and walking | no difference in WBV, PV |
| Church et al. | 2002 | 3 days weekly 12 week long | 7 female, 16 male patients with CAD underwent CR | 30-40 minutes moderate intensity exercise | hematocrit did not change, WBV and PV decreased |
| Lee et al. | 2005 | before and after exercise | 53 patients with CAD | incremental shuttle walk test | increased PV and fibrinogen |

Publications relating to hemorheological alterations induced by long-term exercise training programs in patients with cardiovascular diseases.
